# The Psychological and Physical Benefits of Progressive Muscle Relaxation in Chronic Respiratory Diseases: A Systematic Review

**DOI:** 10.3390/medicina61061055

**Published:** 2025-06-07

**Authors:** Adelina Maritescu, Alexandru Florian Crisan, Camelia Corina Pescaru, Cristian Oancea, Daniela Iacob

**Affiliations:** 1 Doctoral School, “Victor Babes” University of Medicine and Pharmacy Timisoara, Eftimie Murgu Square 2, 300041 Timisoara, Romania; adelina.maritescu@umft.ro; 2 Pulmonary Rehabilitation Center, Clinical Hospital of Infectious Diseases and Pulmonology “Victor Babes”, Gheorghe Adam Street 13, 300310 Timisoara, Romania; pescaru.camelia@umft.ro; 3 Research Center for the Assessment of Human Motion, Functionality and Disability (CEMFD), “Victor Babes” University of Medicine and Pharmacy Timisoara, Eftimie Murgu Square 2, 300041 Timisoara, Romania; 4 Center for Research and Innovation in Personalized Medicine of Respiratory Diseases (CRIPMRD), “Victor Babes” University of Medicine and Pharmacy Timisoara, Eftimie Murgu Square 2, 300041 Timisoara, Romania; oancea@umft.ro; 5 Pulmonology Clinic, Clinical Hospital of Infectious Diseases and Pulmonology “Victor Babes”, Gheorghe Adam Street 13, 300310 Timisoara, Romania; 6 Research Center for Pharmaco-Toxicological Evaluations, ‘Victor Babes’ University of Medicine and Pharmacy Timisoara, Eftimie Murgu Square No. 2, 300041 Timisoara, Romania; iacob.daniela@umft.ro; 7 Department of Neonatology, ‘Victor Babes’ University of Medicine and Pharmacy Timisoara, Eftimie Murgu Square No. 2, 300041 Timisoara, Romania

**Keywords:** progressive muscle relaxation, COPD, cystic fibrosis, COVID-19

## Abstract

*Background and Objectives:* Chronic respiratory diseases, such as COPD, cystic fibrosis, and post-COVID-19, are frequently accompanied by psychological distress and physical impairment. As a non-pharmacological intervention, progressive muscle relaxation (PMR) may benefit these patients psychologically and physiologically. This systematic review aimed to evaluate the effects of PMR on anxiety, depression, fatigue, sleep quality, dyspnea, and pulmonary function in patients with COPD, CF, and COVID-19. *Materials and Methods*: Following PRISMA guidelines, a comprehensive search was conducted across PubMed, Scopus, Web of Science, MEDLINE, Cochrane, SpringerLink, and ClinicalTrials.gov. Eligible studies assessed PMR in adult patients with COPD, CF, or COVID-19. Psychological and physical outcomes were extracted, and methodological quality and risk of bias were evaluated using standardized tools. *Results*: A total of 32 studies were included in the analysis. PMR was consistently associated with reductions in anxiety, depression, fatigue, and sleep-related distress, particularly in patients with COPD and COVID-19. Some also reported improvements in dyspnea and mild pulmonary function tests, but these were more variable. Only one study evaluated PMR in patients with cystic fibrosis, providing the first clinical data for this group. Interventions were predominantly short-term, with significant variation in design, duration, and methodology, and the risk of bias was often moderate or high. *Conclusions*: PMR is a helpful strategy in treating chronic respiratory diseases, particularly for reducing psychological distress and improving sleep. However, the evidence is limited by methodological variations and lack of long-term follow-up. Rigorous research is needed to support clinical application, particularly in cystic fibrosis.

## 1. Introduction

Chronic respiratory diseases, such as Chronic Obstructive Pulmonary Disease (COPD) and Cystic Fibrosis (CF), together with the long-term effects of COVID-19 infection, pose a significant burden on patients and healthcare systems globally [1]. These conditions are characterized by progressive deterioration of lung function, persistent dyspnea, and a considerable reduction in patients’ quality of life. In addition, the psychological impact associated with these diseases, including anxiety, depression, and chronic stress, contributes to worsening symptoms and decreased adherence to treatment. In this context, therapeutic strategies that target both the physical and psychological aspects of chronic respiratory diseases are becoming increasingly important to improve patient care [2].

Progressive muscle relaxation (PMR) is a non-pharmacological technique for reducing stress and improving well-being [3]. Initially developed by Edmund Jacobson in the 1920s, this method involves the controlled tensing and relaxation of different muscle groups to reduce muscle tension and promote an overall state of relaxation [3]. Previous studies have shown that PMR can have beneficial effects on both psychological components, such as reducing anxiety and depression, and physical symptoms, such as improving dyspnea and improving lung function [4,5,6]. In addition, this technique is easy to implement, does not require special equipment, and can be practiced in both clinical settings and at home, providing patients with an effective way to manage their symptoms [7].

Over the years, numerous studies have examined the benefits of PMR in various chronic conditions, but its applicability to patients with COPD and COVID-19 requires greater attention. Patients with COPD often experience severe respiratory difficulties, which limit their daily activities and contribute to decreased functional independence. In the case of COVID-19 and post-COVID syndrome, the effects on the respiratory system can be long-lasting, including a reduction in lung capacity and increased anxiety levels caused by the uncertainty of the disease’s evolution [8].

Regarding CF, a literature review indicates that only one study evaluates the effects of PMR on this condition [1]. As a genetic disease characterized by excessive mucus accumulation in the lungs and recurrent respiratory infections, CF requires complex therapeutic approaches, and the role of muscle relaxation techniques in this pathology remains insufficiently explored. Therefore, this systematic review will contribute to highlighting this gap in the literature and emphasize the need for further studies evaluating the impact of PMR in CF.

Therefore, the analysis of the effects of PMR on patients with COPD and COVID-19 is essential to determine whether this technique can improve respiratory and psychological symptoms associated with these diseases. This systematic review aims to evaluate PMR’s psychological and physical benefits in these conditions. It provides a detailed analysis of existing studies and highlights potential clinical implications for their management. Investigating the mechanisms by which PMR can influence sleep quality, reduce stress levels, and improve patients’ quality of life could contribute to developing more effective and personalized therapeutic strategies for these categories of patients.

## 2. Materials and Methods

This systematic review follows the PRISMA (Preferred Reporting Items for Systematic Reviews and Meta-Analyses) guidelines and was registered in the PROSPERO (CRD420251042624) registry. Existing studies on the effects of progressive muscle relaxation on patients with COPD, cystic fibrosis, and COVID-19 were reviewed. In addition, relevant systematic reviews were included to contextualize the results and highlight any gaps in the literature. These studies were examined to analyze the methodology, data sources, and conclusions drawn, thus avoiding redundancy and ensuring a clear synthesis of the existing evidence.

We referred to the following research computer databases: PubMed, Scopus, Web of Science, MEDLINE, Cochrane, SpringerLink, and ClinicalTrials.gov. Search terms such as “Progressive Muscle Relaxation”, “Chronic Obstructive Pulmonary Disease”, “Cystic Fibrosis”, “COVID-19”, “Anxiety”, “Depression”, “Dyspnea”, “Pulmonary Function”, and ”Chronic Disease”, combined with logical operators (AND, OR) were used to ensure complete coverage of relevant studies. Inclusion criteria targeted studies that investigated the effects of PMR training on psychological symptoms, such as anxiety, depression, and stress, as well as physical symptoms, including dyspnea, lung function, fatigue, sleep quality, and quality of life, among patients diagnosed with COPD, CF, COVID-19, asthma, chronic respiratory diseases, pulmonary arterial hypertension (PAH), lung neoplasm, lung resection, or who had undergone thoracic surgery. We included studies that used PMR as the primary intervention or as part of a treatment program to improve respiratory or psychological symptoms. Randomized controlled trials (RCTs), observational studies, experimental studies, and relevant systematic reviews were also accepted, and the publications included were those available in English and published in the last 10 years.

Exclusion criteria eliminated studies that did not provide distinct data on PMR applied to patients with COPD, CF, COVID-19, asthma, chronic respiratory diseases, PAH, lung neoplasm, or lung resection, or those who had undergone thoracic surgery. Studies that did not directly assess PMR on psychological or physical symptoms associated with these conditions were excluded, as were systematic reviews that did not include studies relevant to the purpose of this analysis. Papers published as conference abstracts, editorials, or opinion pieces were also excluded.

The methodological quality of the included studies was assessed using the critical appraisal tools developed by the Joanna Briggs Institute (JBI) and were appropriate for each type of study (randomized studies, quasi-experimental studies, observational studies, etc.) [9]. Each article was analyzed according to the specific criteria and received an overall score, calculated as the ratio between the “Yes” answers and the total number of applicable questions. Studies were also classified according to the risk of bias (low, moderate, high) based on the answers to the key items in each tool.

Two reviewers carried out independent assessments, and any discrepancies were resolved by discussion and consensus.

In this systematic review, we observed significant variability in the instruments used to assess sleep quality, fatigue, and psychological symptoms. However, most studies used validated standardized scales. For sleep quality assessment, the most commonly used instrument was the PSQI, followed by the SRSS, and the Richards-Campbell Sleep Questionnaire. Fatigue was predominantly assessed with the FSS, CAFS, or the FACIT-T. Anxiety and depression symptoms were measured by instruments such as the HADS, STAI, PHQ-9, BAI, or GHQ-12. Although the methods were not uniform across studies, the above-mentioned were the most commonly used, reflecting an acceptable level of standardization in assessing the effects of PMR.

In this analysis, the term “chronic respiratory disease” also includes conditions with major pulmonary impact, such as lung cancer or post-thoracic surgical recovery, as long as the studies assessed the effects of PMR on respiratory or psychological symptoms.

## 3. Results

After searching the literature using specific keywords, 76 studies were identified. After applying the inclusion criteria, 35 studies investigating the use of PMR techniques in various respiratory conditions were selected. Of these, 14 studies focused on COPD, 10 studies examined the effects of PMR in COVID-19, two studies on asthma, one study on chronic diseases not otherwise specified, one study on PAH, one study on lung cancer, one study on lung resection, one study on thoracic surgery, and one study on CF. In addition, three systematic reviews were included: one on COVID-19, one on COPD, and one on chronic diseases. Of the 14 studies on COPD, four were experimental studies, including randomized clinical trials and pre-post intervention studies, and the remainder were observational studies.

**Figure 1:**
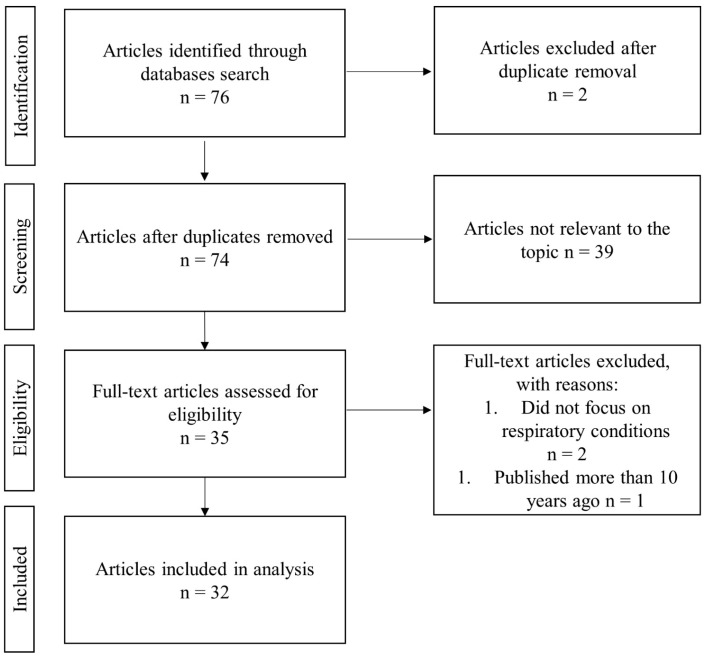
Flowchart of the selection process.

At the end of our selection process, 32 studies were included in this review (see Figure 1). After analyzing the types of studies included in this review, it was found that 9.68% of the articles were reviews (three articles), and 3.23% were observational studies (one article). Experimental studies, including randomized controlled trials (RCTs), pre-experimental, true-experimental, quasi-experimental, and intervention studies, represent 32.26% of the total (11 articles). Also, 54.84% of the articles were classified as original research articles [9], highlighting a predominance of original research in this analysis. To assess the quality of the included studies and the risk of bias, we used the JBI Critical Appraisal Checklist for Quasi-Experimental Studies. The tool assesses aspects such as participant selection, clarity of intervention description, validity of outcome measurement, and adequacy of statistical methods. Each criterion was scored as “Yes” (low risk), “No” (high risk), or “Unclear,” and the overall score was used to classify studies as low, moderate, or high risk. The results are presented in Table 1.

**The effects of PMR on sleep quality in COPD**

Numerous studies have investigated the impact of PMR on sleep quality in patients diagnosed with COPD, highlighting significant investigations of different sleep parameters.

Thus, Kumar et al. [10] conducted a quasi-experimental study that evaluated the effects of PMR on sleep quality in 60 hospitalized patients with COPD. The intervention consisted of performing PMR for 25 min, twice daily, for five consecutive days. The results revealed a significant increase in sleep quality, reflected by the reduction in the mean PSQI score from 10.80 (SD = 3.72) to 4.0 (SD = 1.72) (*p* < 0.05). These data support the effectiveness of PMR as a non-pharmacological intervention that is accessible and without additional costs for health systems in improving sleep quality in patients with COPD.

In another study, Pedramrazi et al. [14] explored the effects of controlled breathing exercises, including PLB, diaphragmatic breathing, and controlled coughing, on sleep quality in 64 patients with COPD. Over seven weeks, the intervention group experienced a significant decrease in PSQI score from 11 to 7 (*p* < 0.001), highlighting the importance of integrating controlled breathing techniques into COPD management programs to reduce associated sleep disturbances.

Mujahid et al. [24] also conducted a randomized clinical trial comparing the effects of Jacobson’s PMR with Laura Mitchell’s relaxation technique on sleep quality and quality of life in patients with COPD stages III and IV. The study, which included 68 participants, demonstrated that both techniques improve sleep quality; however, the Laura Mitchell method had a greater impact on general clinical parameters. These results suggest the need to systematically integrate relaxation techniques into pulmonary rehabilitation programs to maximize therapeutic benefits for patients with COPD.

**PMR and PLB reduce fatigue and depression in COPD**

The results of the studies analyzed indicate that PMR combined with PLB has significant effects on fatigue and depression in patients with COPD. The study conducted by Patimah et al. [12] demonstrated a considerable reduction in the fatigue and depression score in the intervention group compared to the control group, with a *p*-value < 0.001. This suggests that PMR and PLB may be an effective strategy for improving patients’ emotional state and comfort with COPD. However, the study did not reveal significant changes in lung function, with a *p*-value = 0.191, which suggests that the effects of this technique are instead on the psychological and physical components related to fatigue rather than on respiratory function.

The results obtained by Danismaya et al. [17] are consistent with those of the previous study, showing a significant decrease in the fatigue and depression score after applying PMR and PLB, also with a *p*-value < 0.001. Also, similar to the study by Patimah et al. [12], no improvement in lung function was identified, with a *p*-value of 0.191. The author suggests that PMR and PLB can be integrated into pulmonary rehabilitation and care plans for patients with COPD, as they are practical interventions for reducing fatigue and depression without directly impacting respiratory function.

**PMR enhanced oxygen saturation in COPD**

The study by Widiastuti et al. [13] showed that PMR influences oxygen saturation in patients with COPD. The study used a pretest-posttest design to analyze changes in oxygen saturation before and after the application of PMR. The results showed a significant increase in the mean oxygen saturation value from 88.47% before the intervention to 89.33% after the application of PMR. Statistical analysis using the paired *t*-test indicated a *p*-value of 0.002, which confirms a significant effect of PMR on oxygen saturation.

**Relaxation exercises improve sleep quality and dyspnea in COPD**

The results of the study conducted by Işıkel et al. [15] indicated that relaxation exercises significantly affect dyspnea and sleep quality in patients with COPD. The study was a randomized controlled trial performed on a sample of 67 patients with COPD, of whom 34 were assigned to the intervention group and 33 to the control group. Patients in the intervention group practiced relaxation exercises for six weeks, while patients in the control group performed only breathing exercises.

The results showed a significant reduction in the severity of dyspnea in the intervention group, with substantial decreases in the scores on the Modified Borg Scale (MBS) and Modified Medical Research Council (mMRC) scales (*p* < 0.001). There was also a significant improvement in the global Pittsburgh Sleep Quality Index (PSQI) score (*p* < 0.001), indicating an increase in sleep quality. Several subscales of the PSQI showed significant improvements, including subjective sleep quality (*p* < 0.001), sleep latency (*p* = 0.029), sleep duration (*p* < 0.001), sleep efficiency (*p* = 0.047), and daytime dysfunction (*p* < 0.001). These changes suggest that relaxation exercises can reduce sleep disturbances and improve daytime fatigue in patients with COPD.

**PMR reduces fatigue and enhances sleep quality in COPD**

The reviewed studies provide quantifiable data on the effects of PMR exercises on fatigue, sleep quality, and dyspnea in patients with COPD.

Şahin et al. [16] found that PMR resulted in a significant decrease in the fatigue score measured by the COPD and Asthma Fatigue Scale (CAFS), from 57.35 ± 9.88 before the intervention to 23.56 ± 6.54 after the application of PMR (*p* < 0.001). Global sleep quality was also improved, with the global PSQI score decreasing from 9.52 ± 0.22 to 5.35 ± 0.54 (*p* < 0.001). The PSQI subscales showed notable improvements: sleep latency decreased from 2.89 ± 1.78 to 1.08 ± 0.54 (*p* < 0.001), sleep duration increased from 2.47 ± 1.54 to 1.22 ± 0.36 (*p* < 0.05), and sleep efficiency was significantly improved (*p* < 0.05).

In a randomized controlled trial with 91 patients, Chegeni et al. [20] confirmed that PMR led to a significant reduction in fatigue, with the Fatigue Severity Scale (FSS) score decreasing from 46.75 ± 14.60 to 24.66 ± 16.17 (*p* < 0.001) in the intervention group, while the control group showed no significant changes. Also, analysis of sleep components indicated significant improvements in subjective sleep quality (from 1.20 ± 0.72 to 0.53 ± 0.54, *p* < 0.001), sleep latency (from 1.60 ± 1.26 to 0.82 ± 0.93, *p* < 0.001), and sleep duration (from 0.87 ± 0.82 to 1.77 ± 0.92, *p* < 0.001).

Yilmaz et al. [22] observed that patients who practiced PMR significantly reduced fatigue as measured by CAFS, with scores decreasing from 70.77 ± 16.22 in the control group to 42.76 ± 14.37 in the intervention group (*p* = 0.001). Also, sleep quality, as measured by the COPD and Asthma Sleep Impact Scale (CASIS), significantly improved in the intervention group, with scores decreasing from 64.91 ± 16.20 to 35.29 ± 13.80 (*p* < 0.001). Patients in the intervention group showed a significant reduction in dyspnea as measured by the Medical Research Council Dyspnea Scale (*p* < 0.05).

**PMR and PLB reduce dyspnea in COPD**

The results of the study conducted by Massie et al. [18] showed that the combined use of the PLB and PMR techniques resulted in significant changes in the severity of dyspnea in patients with COPD. The study included two groups of 20 patients each, one of which performed both PLB and PMR and the other only PLB.

In the group that performed both PLB and PMR, the severity of dyspnea decreased from 75% of patients with severe dyspnea before the intervention to 10% after the intervention. In comparison, the percentage of those with mild dyspnea increased from 25% to 90% (*p* = 0.000). In the group that performed only PLB, the severity of dyspnea changed less, with the percentage of patients with severe dyspnea decreasing from 50% to 45% and those with mild dyspnea increasing from 50% to 55% (*p* = 1.000). Comparing the two groups, the differences in the reduction of the severity of dyspnea were statistically significant (*p* = 0.034) [18].

Statistical analysis did not identify a significant correlation between the degree of dyspnea and factors such as smoking (*p* = 1.000), history of lung infections (*p* = 1.000), or use of pharmacological therapy (*p* = 0.70). Previous exposure to irritants or pollutants was associated with a higher degree of dyspnea in the group that performed PLB alone (*p* = 0.005) [18].

**Device-guided breathing improved dyspnea and respiratory pattern in COPD**

The study conducted by Borge et al. [19] showed that GDB-guided (device-guided breathing) deep breathing significantly affects dyspnea, breathing patterns, and quality of life in patients with COPD. The study included 150 patients with moderate to severe COPD, who were divided into three groups: one who used GDB, one who listened to music (MLG), and one control, where the patients sat at rest (SSG).

After four weeks, patients in the GDB group reported a significant reduction in dyspnea measured by Global Rating Change (GRC) (*p* = 0.03), with a persistent effect at four months compared to the group that listened to music (*p* = 0.04), but without a significant difference compared to the control group. Regarding respiratory rate (RR), a significant decrease was observed in the GDB group (*p* < 0.001) at four weeks. Also, the St George’s Respiratory Questionnaire (SGRQ) symptom score showed significant improvements in all groups (*p* < 0.05–0.01) [19].

Breathing analysis showed a significant decrease in respiratory rate in the GDB group, from 16 ± 5.1 to 13.4 ± 5.6 at the beginning of the sessions and from 11.8 ± 5.1 to 9 ± 5 at the end of the sessions (*p* < 0.01). In contrast, the MLG and SSG groups did not show significant changes. Inspiratory time (TIN) increased from 1.5 ± 0.5 to 1.9 ± 1.1 (*p* < 0.01) in the GDB group, and expiratory time (TEX) increased from 2.7 ± 1.3 to 3.4 ± 1.6 (*p* < 0.05), indicating an improvement in respiratory control [19].

SGRQ symptom scores decreased in the GDB group, from 57.4 ± 21.8 to 52.1 ± 21.9 (*p* < 0.05), and similar reductions were seen in the MLG and SSG groups. However, there were no significant differences between groups in terms of impact on quality of life (*p* > 0.05) [19].

**PMR enhances FEV_1_, reduces anxiety and depression, and improves quality of life in COPD.**

The study’s results by Volpato et al. [21] indicated that relaxation techniques positively affected respiratory function and the psychological state of patients with COPD. The meta-analysis included 25 RCTs and evaluated respiratory and psychological parameters.

Regarding pulmonary function, relaxation techniques had a small but positive effect on the FEV_1_ percentage value, with an effect size of d = 0.20 (95% CI: 0.40–−0.01). Regarding anxiety and depression, a significant reduction in both symptoms was observed after the application of relaxation techniques. Anxiety scores had an effect size of d = 0.26 (95% CI: 0.42–0.10), and those for depression showed an effect of d = 0.33 (95% CI: 0.53–0.13) [21].

The largest effect size was observed in quality of life, where relaxation techniques had a positive impact with d = 0.38 (95% CI: 0.51–0.24) [21].

The overall quality of the included studies was assessed as medium/high, according to the PEDro scale [21].

**PMR enhanced treatment adherence in COPD**

The results of the study by Mujahid et al. [24] indicated that Jacobson’s Progressive Relaxation Technique (JPRT) and Laura Mitchell’s Relaxation Technique (LMRT) significantly affected sleep quality and quality of life in patients with COPD. The study was a randomized clinical trial, including 68 patients, divided into two groups: group A, treated with LMRT, and group B, treated with JPRT. The interventions were applied five times weekly for 2 weeks.

The results showed that the mean sleep quality score, measured by the PSQI, decreased significantly in group A, from 13.5 ± 2.46 to 4.10 ± 1.26 (*p* = 0.001), and in group B, from 13.80 ± 2.35 to 5.3 ± 1.41 (*p* = 0.001) [24].

Regarding quality of life, measured by the St. George’s Respiratory Questionnaire (SGRQ), the initial score was similar between the two groups (69.70 ± 4.8 for group A and 69.76 ± 2.09 for group B, *p* = 0.885). After the intervention, group A recorded a reduction in the score to 22.83 ± 1.23, while group B had a smaller reduction, to 36.00 ± 6.5 (*p* = 0.000) [24].

Statistical analysis showed significant improvements in both groups. Still, the Laura Mitchell technique (LMRT) had a more pronounced effect on the quality of sleep and life of patients compared to the Jacobson technique (JPRT) [24].

**PMR reduces anxiety and enhances quality of life in COVID-19**

Xiao et al. [29] conducted an observational study of 79 COVID-19 patients admitted to a hospital in Wuhan. Patients were divided into an intervention group and a control group. Those in the intervention group practiced PMR for 30 min daily for 5 days. Anxiety scores decreased from 14.2 ± 3.5 to 7.6 ± 2.8 (*p* < 0.05), depression from 12.8 ± 3.1 to 6.3 ± 2.5 (*p* < 0.05), and sleep quality according to the PSQI improved (from 10.5 ± 2.7 to 5.2 ± 1.9; *p* < 0.05).

Liu et al. [30] conducted a randomized controlled trial of 51 COVID-19 patients. The intervention group practiced PMR for 30 min per day for 5 days. STAI anxiety score decreased significantly (from 55.3 ± 4.1 to 38.6 ± 3.9; *p* < 0.001), and sleep quality according to SRSS improved (from 32.4 ± 5.7 to 21.9 ± 4.2; *p* < 0.001).

Seid et al. [28] conducted a meta-analysis of four studies totaling 227 patients with COVID-19. Results indicated a significant anxiety reduction (SMD: −1.35; 95% CI: −2.38, −0.32; *p* = 0.01), an improvement in depression (SMD: −1.12; 95% CI: −2.01, −0.45; *p* = 0.03), and an increase in quality of life (SMD: 1.04; 95% CI: 0.38, 1.57; *p* = 0.02).

Saleh et al. [26] conducted a randomized controlled trial on 146 COVID-19 patients from a private hospital in Jordan. The experimental group practiced PMR for 30 min per day for 5 days, and the control group received only standard care. The mean anxiety score (Spielberg State-Trait Anxiety Scale) was significantly reduced (from 57.3 ± 8.1 to 40.6 ± 7.4; *p* < 0.05). Sleep quality, as measured by the Sleep State Self-Rating Scale (SRSS), increased significantly (*p* < 0.05).

Özlü et al. [25] conducted a randomized controlled trial on 67 COVID-19 patients (33 in the experimental and 34 in the control group). The intervention group performed progressive muscle relaxation (PMR) exercises twice daily for 5 days. The anxiety score (State Anxiety Scale) decreased significantly in the experimental group (*p* < 0.05), while no significant changes were observed in the control group. Sleep quality was also improved according to the Richard-Campbell Sleep Questionnaire (*p* < 0.05).

Ganjeali et al. [31] analyzed the effects of PMR on stress and anxiety among nurses caring for COVID-19 patients. The study was conducted on 46 nurses. After the intervention, stress levels decreased from 13.91 ± 2.41 to 10.95 ± 2.01 (*p* < 0.001), and anxiety from 13.34 ± 3.41 to 9.47 ± 2.37 (*p* < 0.001).

Herdiman et al. [27] investigated the effects of PMR on anxiety in 40 hospitalized COVID-19 patients in Bandung. In total, 20 patients practiced PMR for 20–30 min twice daily for 5 days. The results showed a significant reduction in anxiety (from 29.7 ± 5.6 to 18.4 ± 4.2; *p* < 0.0001) and an improvement in sleep quality (PSQI from 11.3 ± 2.9 to 6.8 ± 2.1; *p* < 0.0001).

**PMR and PR improve anxiety, sleep quality, and mental health in COVID-19**

Maritescu et al. [4] found that patients who performed both PR and PMR for 21 days had significant improvements in lung function, with an increase in FEV_1_ of 8.9% in the PR group and 12.4% in the PR + PMR group (*p* < 0.0001). There was also a decrease in the GHQ-12 score by 4.32 points (*p* < 0.0001), a reduction in depressive symptoms measured by the PHQ-9 by 3.92 points (*p* < 0.0001), and a decrease in anxiety (GAD-7) by 3.74 points (*p* < 0.0001). Sleep quality was improved, with a decreased PSQI score of 4.01 points (*p* < 0.0001). Regarding exercise capacity, the 6 min walk test (6MWT) showed an increase of +43.6 m in the PR group and +47.2 m in the PR + PMR group, with no significant difference between groups (*p* = 0.089).

Hajibashi et al. 26] found that patients who received pulmonary telerehabilitation (PTR) combined with PMR for 6 weeks experienced an improvement in sleep quality of 3.21 points (*p* = 0.001, 95% CI: 1.20–4.09), a reduction in fatigue of 2.87 points (*p* = 0.041, 95% CI: 4.79–5.25), and a decrease in anxiety of 3.05 points (*p* = 0.001, 95% CI: 1.21–4.47) compared with the group that received PTR alone. No significant differences were observed between groups for oxygen saturation levels (*p* = 0.312) or lung capacity (*p* = 0.087).

**PMR improves sleep quality, emotional state, and quality of life in patients with CF**

The RCT study conducted by Maritescu et al. [32] investigated the effects of the PMR technique, applied three times a week for four weeks, in a group of 22 adult patients diagnosed with cystic fibrosis integrated into a standard pulmonary rehabilitation program. The experimental group (*n* = 11), which additionally followed the PMR technique, was compared with a control group (*n* = 11), which followed only the standard program.

The results indicated significant improvements in perceived psychological and functional parameters. At the end of the intervention, the mean HADS-A score for anxiety decreased from 9.45 ± 3.11 to 6.82 ± 2.35 (*p* = 0.05), and the HADS-D score for depression decreased from 8.36 ± 2.94 to 5.91 ± 2.16 (*p* = 0.02) in the group that practiced PMR. These differences were not observed in the control group, where values remained relatively constant [32].

Sleep quality, assessed by the PSQI, improved significantly in the experimental group, with a decrease in the total score from 8.64 ± 2.72 to 5.27 ± 1.83 (*p* < 0.01), suggesting a substantial reduction in sleep disturbances. The control group did not show significant changes (*p* > 0.05) [32].

The CFQ-R questionnaire also showed a significant increase in scores in the emotional functioning domain, from 58.6 ± 11.9 to 69.1 ± 10.3 (*p* = 0.01), and an improvement in the general perception of health, with an increase from 62.8 ± 12.1 to 72.3 ± 9.5 (*p* = 0.02) [32].

**PMR reduces anxiety in asthma**

Dewi et al. [33] conducted a pre-experimental study on 26 patients with acute asthma attacks admitted to an emergency department. The assessment was performed using the Beck Anxiety Inventory (BAI) before and after a 10 min PMR session and applied 10 min after the presentation. The results indicated a decrease in severe anxiety from 46.15% to 0% and an increase in cases with mild anxiety from 11.54% to 57.69%. The differences were statistically significant (*p* = 0.001), suggesting the effectiveness of PMR in controlling acute anxiety in asthma.

**PMR improves perceived stress and quality of life in asthma patients**

Georga et al. [34] conducted a randomized controlled trial in 42 patients with mild intermittent or chronic asthma. The intervention consisted of an 8-week stress management program, including PMR and biofeedback-assisted breathing. The results showed a significant decrease in the PSS (Perceived Stress Scale) score from 21.10 ± 2.3 to 14.23 ± 2.7 (*p* < 0.0001), an increase in the asthma control score (ACQ), and a significant improvement in the quality of life (AQLQ). All results were statistically significant (*p* < 0.01).

**PMR improves sleep quality in patients with chronic diseases**

A literature review by Mirzanah et al. [35], which included six studies on the effects of PMR on sleep quality in patients with chronic diseases, showed that all studies reported significant improvements in PSQI (Pittsburgh Sleep Quality Index). For example, PSQI scores decreased, on average, from 10.5 ± 2.4 to 5.8 ± 1.7 following the application of PMR. This supports PMR as an effective and affordable method of managing sleep disorders in chronic pathologies.

**PMR reduces anxiety and depression in PAH**

Li et al. [36] included 130 patients with PAH in a randomized trial in which the intervention group underwent group and individual PMR for 12 weeks. The mean anxiety score (SAS) decreased from 58.1 ± 5.6 to 49.7 ± 4.8, and the depression score (SDS) from 62.9 ± 5.1 to 50.5 ± 4.9 (*p* < 0.01). In addition, the mental domain scores of the SF-36 increased significantly, demonstrating an improvement in quality of life.

**PMR improves oxygen saturation and relaxation in lung cancer patients**

The study by Dinaryanti et al. [37] included 19 lung cancer patients who applied PMR combined with PLB twice daily for 6 days. The mean oxygen saturation increased from 92.3% ± 1.8 to 94.7% ± 1.5, and the mean perceived relaxation score increased by 2.1 points on a visual analog scale (*p* < 0.05). The results support the effectiveness of PMR in improving symptoms of dyspnea and emotional distress.

**PMR accelerates postoperative recovery after lung resection**

Temel Aksu et al. [38] conducted an RCT on 26 patients undergoing lung resection, applying PMR twice daily for 7 days postoperatively. The PSQI score decreased from 9.2 ± 1.3 to 4.8 ± 1.0, and the SF-36 quality of life score increased by 12.6 points in the intervention group (*p* < 0.05). The intensity of postoperative pain was significantly reduced compared to the control group, supporting the role of PMR in improving recovery after thoracic interventions.

**PMR supports pulmonary rehabilitation after thoracic surgery**

Lin et al. [39] evaluated 140 patients undergoing major thoracic surgery, divided into two equal groups. The intervention group received personalized PMR based on respiratory muscle assessment for 7 days. Results indicated an increase in FVC from 2.29 ± 0.21 L to 2.68 ± 0.26 L, an improvement in FEV_1_ from 1.93 ± 0.18 L to 2.23 ± 0.22 L, and a significant reduction in bronchial obstruction (*p* < 0.05). In addition, patients in the PMR group had more efficient secretion clearance and higher quality of life scores.

The characteristics of the study population vary considerably between studies, including patients with COPD, lung cancer, asthma, and postoperative thoracic syndrome. However, most studies do not report details about associated cardiovascular or metabolic comorbidities, such as heart failure, obesity, sleep apnea syndrome, or pulmonary hypertension; these factors may influence the level of sympathetic activation, dyspnea, and response to progressive muscle relaxation therapy. The absence of this information limits the ability to tailor the intervention to distinct clinical subgroups. Future research should investigate the impact of these comorbidities on the effectiveness of PMR to support a more personalized implementation in clinical practice.

## 4. Discussion

The collective findings of this systematic review underscore the therapeutic utility of PMR as a non-pharmacological intervention for alleviating psychological and physiological symptomatology in individuals with chronic respiratory disorders. A substantial proportion of the included studies demonstrated clinically meaningful reductions in anxiety, depressive symptoms, perceived fatigue, and sleep disturbances, particularly among patients with COPD and those exhibiting post-acute sequelae of COVID-19 infection. Nonetheless, the evidence remains heterogeneous concerning objective pulmonary function outcomes, and data on less extensively studied conditions—such as cystic fibrosis, pulmonary arterial hypertension, and thoracic malignancies—are currently limited and preliminary.

Studies with positive results on psychological symptoms indicate well-defined physiological mechanisms. By alternating muscle contraction and relaxation, PMR reduces sympathetic nervous system activation and increases parasympathetic tone, evidenced by decreased heart rate, blood pressure, and salivary cortisol levels [15]. These autonomic changes induce a state of physiological calm favorable to reduced levels of anxiety and depression. In studies of COVID-19 patients, PMR produced rapid improvements in STAI and HADS scores, even after only 5–7 days of application [32].

From a psychological standpoint, PMR has been associated with heightened somatic awareness and enhanced interoceptive accuracy. By focusing on the sustained cognitive cycle of muscle tension and relaxation, PMR helps individuals identify, modulate, and reduce their manifestations of stress and bodily discomfort. This mechanism is essential in chronic respiratory diseases, where dyspnea is often exacerbated by anxiety. Thus, the technique contributes not only to reducing the stress response but also to the perceptual reinterpretation of the physiological symptoms associated with the disease [39]. In multiple studies included in this review, PMR has been shown to be effective in improving sleep quality, both in patients with COPD and in those undergoing treatment for lung cancer [20]. This effect is supported by evidence indicating the involvement of neurophysiological mechanisms, such as inhibition of the ascending reticular activating system (ARAS), responsible for the state of alertness. By reducing sympathetic activity and stimulating parasympathetic activity, PMR decreases global neuronal excitability, facilitating sleep initiation and prolonging deep NREM sleep phases [5,16,20] Additionally, PMR relieves daytime muscle tension, especially in areas such as the neck, shoulders, and chest—regions that are often tense in people with chronic respiratory disorders. Relaxing these muscle groups can help reduce nighttime dyspnea and improve respiratory mechanics [7,15]. Additionally, some authors have postulated that PMR may enhance endogenous melatonin synthesis and entrain circadian rhythm stability via modulation of pineal gland activity [39].

Moreover, empirical investigations conducted by Akgün Şahin and Işıkel have demonstrated that PMR is associated with a measurable reduction in sleep onset latency and a decreased frequency of nocturnal awakenings, culminating in substantial enhancements in overall sleep efficiency. These outcomes appear to be biologically mediated by attenuated hypothalamic-pituitary-adrenal (HPA) axis activation, as evidenced by diminished circulating cortisol concentrations alongside elevated nocturnal heart rate variability—both of which serve as established biomarkers of parasympathetic dominance and physiological restoration during sleep [15,39].

Related to fatigue and affective regulation, empirical data suggest that interventions integrating PMR with respiratory modulation techniques, such as PLB, yield superior outcomes compared to either modality applied in isolation. Investigations conducted by Patimah et al. and Danismaya et al. have reported substantial declines in both somatic and psychological fatigue, along with attenuated depressive symptomatology, despite the absence of statistically significant improvements in spirometric indices such as FEV_1_ or peripheral oxygen saturation (SpO_2_) [12,17].

These findings lend credence to the hypothesis that the therapeutic efficacy of PMR is primarily perceptual and experiential rather than contingent upon quantifiable structural alterations in pulmonary mechanics. By mitigating sensations of exhaustion and emotional distress, PMR may enhance functional capacity and promote adherence to rehabilitative regimens. These outcomes are further corroborated by recent advances in affective neuroscience, which demonstrate that mind-body relaxation practices—including PMR—engage and modulate neural circuits encompassing the amygdala, medial prefrontal cortex, and hypothalamic structures, all of which play integral roles in emotional homeostasis, stress appraisal, and illness perception [15].

Additionally, findings from Lin et al. suggest that therapist-guided relaxation protocols may attenuate postoperative fatigue and facilitate airway clearance in individuals undergoing thoracic surgical procedures, thereby implying a potential indirect enhancement of respiratory mechanics through the alleviation of thoracic muscular tension and augmentation of chest wall mobility [39].

This evidence emphasizes the importance of applying PMR in a guided, repetitive setting and, if possible, in combination with other respiratory interventions. Massie et al. [18] showed that the combined application of PMR and PLB significantly reduced dyspnea scores and improved exercise tolerance, even without significant spirometric changes. However, not all results have been positive. Studies with negative or inconsistent results, such as those by Patimah or Mujahid, indicate methodological problems, such as insufficient intervention duration, lack of therapeutic guidance, or small sample sizes [12,24]. Also, in studies in which PMR was self-administered, without professional supervision, the effectiveness was lower, suggesting that therapist involvement is essential for the success of the intervention.

In complement to the mechanisms mentioned above, a growing body of literature has begun to elucidate additional physiological pathways through which PMR may exert its therapeutic influence. These include enhanced heart rate variability (HRV), a surrogate marker of parasympathetic reactivity and autonomic flexibility [15], as well as increased activation of the medial prefrontal cortex, a region implicated in affect regulation and executive emotional control [3]. Furthermore, reductions in circulating IL-6 and other proinflammatory cytokines have been observed, suggesting a plausible anti-inflammatory cascade indirectly initiated by PMR engagement [39].

However, these promising insights and mechanisms remain insufficiently substantiated, given the limited number of studies employing objective physiological biomarkers such as HRV, electroencephalography (EEG), salivary cortisol, or inflammatory mediators. Most available data derives from subjective self-reported outcomes, which are inherently susceptible to expectancy effects and placebo responses. Additionally, the heterogeneity in PMR implementation, particularly concerning intervention frequency, session duration, and delivery modality (guided versus unguided), poses significant challenges to cross-study comparability and undermines replicability. To date, only a minority of studies (e.g., Lin et al., Aksu et al.) have employed individually tailored, clinician-supervised protocols calibrated to patients’ respiratory capacity, which appear to yield superior therapeutic outcomes [38,39].

Another relevant aspect, but rarely addressed in the included studies, is the influence of concomitant treatments on the response to PMR. The use of chronic oxygen therapy, heart rate-lowering medications (e.g., beta-blockers), or sympathomimetic bronchodilators can alter autonomic nervous system activity, thereby affecting the effectiveness of progressive muscle relaxation. These therapies may influence heart rate variability (HRV), a sensitive marker of the physiological response to relaxation interventions. The absence of these data in the reviewed reports limits the ability to fully understand the mechanisms and effects of PMR, and future research should control for and document these variables.

Literature review suggests that certain patients may benefit more from progressive muscle relaxation therapy. Common features of “responders” include the presence of anxiety associated with the respiratory condition, significant difficulty initiating or maintaining sleep, and an increased level of body awareness. In particular, patients with severe COPD and pronounced neurovegetative symptoms (insomnia, palpitations, chronic muscle tension) appear to respond better to PMR intervention, especially when it is integrated into a multidisciplinary program. However, studies that used longer interventions or daily guided practices have reported more consistent improvements, suggesting an adherence- and training-dependent effect.

A further critical limitation of the existing evidence base is the scarcity of long-term follow-up data. Most included studies assessed outcomes within a narrow temporal window, typically ranging from 5 to 14 days post-intervention, thereby precluding meaningful insights into the durability or sustainability of observed benefits following cessation of PMR practice. Additionally, very few investigations have systematically evaluated patient adherence to PMR in real-world, unsupervised environments—an omission that hinders assessments of feasibility and large-scale implementation potential.

Methodologically, one recurrent shortcoming was the presence of moderate to high risk of bias in 13 out of the 32 included studies. This was predominantly attributable to methodological constraints such as lack of random sequence generation, inadequate assessor blinding, and incomplete reporting of attrition rates. Moreover, small sample sizes (fewer than 40 participants) were noted in over one-third of studies, substantially limiting statistical power and reducing the external validity of findings.

Nevertheless, this review yields several novel contributions to the field. Recent investigations in thoracic surgery cohorts (Aksu, Lin) suggest that PMR may facilitate postoperative recovery by attenuating pain, enhancing ventilatory mechanics, and promoting adequate airway clearance [38,39]. In parallel, the study by Maritescu et al. represents the first known attempt to examine PMR in the context of cystic fibrosis, reporting improvements in subjective sleep quality and perceived stress, which is a promising avenue for future research [32].

Considering the findings and limitations, future research agendas should prioritize the implementation of rigorously designed randomized controlled trials incorporating standardized PMR protocols, extended intervention durations, and robust post-intervention follow-up periods. Crucially, such trials must integrate objective physiological endpoints, including HRV, EEG indices, and salivary cortisol concentrations, with subjective self-reported outcomes to elucidate mechanistic underpinnings and enhance scientific validity.

In parallel, the scalability and accessibility of PMR interventions should be expanded through digital delivery platforms, such as mobile health applications and web-based programs, particularly for patients with limited mobility or access to in-person services. Furthermore, comprehensive cost-effectiveness analyses comparing PMR to alternative psychophysiological interventions (e.g., mindfulness-based stress reduction, biofeedback, or cognitive behavioral therapy) are warranted to inform evidence-based integration into clinical rehabilitation guidelines.

## 5. Conclusions

This systematic review provides converging evidence supporting the clinical utility of PMR as a complementary intervention for individuals with chronic respiratory diseases. Across a diverse array of study designs and patient populations, PMR has consistently demonstrated beneficial effects on psychological distress, sleep disturbances, and fatigue perception. These improvements appear to be mediated primarily through autonomic modulation and enhanced interoceptive awareness rather than through direct alterations in pulmonary function.

While the quality of evidence varies, and limitations such as short follow-up duration and methodological heterogeneity remain salient, the cumulative findings underscore PMR’s potential role as an accessible, non-pharmacological adjunct within pulmonary rehabilitation paradigms. Future investigations employing standardized protocols, objective biomarkers, and longer-term evaluations are warranted to substantiate its physiological mechanisms and optimize its integration into multidisciplinary clinical practice.

## Figures and Tables

**Table 1:** Characteristics, outcomes, and methodological quality of the studies included in the review.

No.	Author (year)	Study Type	Population/Domain	Intervention/Methodology	Results	Conclusions	JBI Score	Risk of Bias
1	Kumar L., et al. (2024) [10]	Quasi-experimental pre-test-post-test control group study	60 hospitalized COPD patients (30 experimental, 30 control)	PMR for 25 min, twice daily for 5 days; sleep quality assessed using PSQI	PMR significantly improved sleep quality in the experimental group; PSQI score reduced from 10.80 (SD = 3.72) to 4.0 (SD = 1.72), *p* < 0.05	PMR is effective in improving sleep quality in hospitalized COPD patients and can be integrated into routine care without additional costs	10/10	Low
2	Eldefrawy M.M., et al. (2023) [11]	Quasi-experimental single-group pretest/post-test study	37 COPD patients in ICU at Cairo University Hospitals	PMR for 20–30 min, twice daily, during ICU stay; sleep quality assessed using PSQI	Significant improvement in sleep quality; reduction in sleep disturbances and improved PSQI scores; reduced sleep disturbances; improved PSQI scores; *p* < 0.05	PMR positively affects sleep problems in COPD patients, reducing sleep disturbances and improving overall sleep quality	6/8	Moderate
3	Patimah, et al. (2017) [12]	Pretest-posttest randomized control group design	COPD patients in the Provincial Hospital of Jayapura	PMR with PLB; evaluation using FACIT-T, BDI II	PMR with PLB significantly reduced fatigue and depression scores (*p* = 0.000); no significant effect on lung function (*p* = 0.191)	PMR with PLB is effective in reducing fatigue and depression in COPD patients and can be part of routine care	6/10	Moderate
4	Widiastuti, et al. (2023) [13]	Pre-experimental one-group pretest-posttest study	21 COPD patients in IRNA C RSUP Sanglah Denpasar	PMR for oxygen saturation improvement; paired *t*-test analysis	PMR significantly improved oxygen saturation (*p* = 0.002)	PMR should be included in hospital COPD care at least twice daily	6/8	Moderate
5	Pedramrazi Y., et al. (2015) [14]	Clinical trial	64 COPD patients divided into experimental and control groups	Controlled breathing techniques, including PLB, diaphragmatic breathing, and coughing exercises for 7 weeks	Significant improvement in sleep quality in the intervention group (*p* < 0.001)	Controlled breathing exercises should be integrated into COPD sleep management programs	6/10	Moderate
6	Isikel, et al. (2023) [15]	Randomized controlled clinical study	67 COPD patients with severe dyspnea	Relaxation exercises at home for 6 weeks	Significant improvement in sleep quality and dyspnea reduction (*p* < 0.001)	Relaxation exercises should be included in standard COPD treatment plans	6/10	Moderate
7	Sahin, et al. (2015) [16]	Single-group pretest/post-test study	45 COPD patients	PMR measured with the Fatigue Severity Scale and Pittsburgh Sleep Quality Index	PMR reduced fatigue and improves sleep quality	PMR should be integrated into COPD rehabilitation nursing care	9/10	Moderate
8	Danismaya, et al. (2024) [17]	Pretest-posttest randomized control group design	COPD patients	PMR with PLB	Significant decrease in fatigue and depression scores (*p* = 0.000); no effect on lung function (*p* = 0.191)	PMR with PLB should be used in COPD care for fatigue and depression management	6/10	Moderate
9	Massie, et al. (2022) [18]	Quasi-experimental pretest-posttest study	40 COPD patients	Combination of PLB and PMR	Significant reduction in dyspnea severity (*p* < 0.05)	A combination of PLB and PMR is beneficial in COPD dyspnea management	9/10	Moderate
10	Borge, et al. (2015) [19]	Double-blind randomized controlled trial	150 COPD patients with moderate to severe symptoms	Guided deep breathing with a device vs. music listening vs. control group	Significant improvement in breathlessness and respiratory pattern in the guided deep breathing group (*p* < 0.03)	Guided deep breathing may be used as a self-management technique in COPD	9/10	Low
11	Chegeni, et al. (2018) [20]	Randomized controlled clinical trial	91 COPD patients (grades 3 and 4)	PMR for 8 weeks; assessed fatigue and sleep quality	PMR decreased fatigue and improved sleep quality subscales (*p* < 0.05), but no improvement in global sleep quality	PMR can be effective in reducing fatigue and improving certain aspects of sleep quality in COPD patients	6/10	Moderate
12	Volpato, et al. (2015) [21]	Systematic review and meta-analysis	25 RCTs on COPD patients (inpatients and outpatients)	Relaxation techniques, including PMR, guided imagery, and breathing control	Small but significant effects on FEV_1_, anxiety, depression, and quality of life	Relaxation training can have moderate benefits on psychological well-being and respiratory function	9/10	Low
13	Yilmaz, et al. (2017) [22]	Randomized controlled experimental study	68 COPD patients	PMR performed at home; measured dyspnea, fatigue, and sleep quality	Significant reductions in dyspnea and fatigue, improved sleep scores (*p* < 0.05)	PMR can be an effective home-based intervention for COPD symptom management	7/10	Moderate
14	Hyland M.E., et al. (2016) [23]	Comparative study	COPD patients	Comparison of six relaxation techniques via DVD	The most preferred technique was ‘thinking of a nice place’ followed by progressive relaxation	Providing multiple relaxation options rather than one may improve adherence	6/8	Low
15	Mujahid H., et al. (2022) [24]	Randomized Clinical Trial	68 COPD patients (stages 3 and 4)	Comparison of Jacobson’s Progressive Relaxation Technique and Laura Mitchell’s Relaxation Technique, assessed via the Pittsburgh Sleep Quality Index and St. George’s Respiratory Questionnaire	PMR significantly improved sleep and quality of life in COPD patients (*p* < 0.001). Pittsburgh Sleep Quality Index (PSQI) score and St. George’s Respiratory Questionnaire (SGRQ) score showed significant reductions post-treatment in both groups (*p* = 0.001, *p* < 0.001).	Both relaxation techniques are effective for COPD patients, but Laura Mitchell’s technique had a more significant impact on quality of lifeand sleep quality	7/10	Moderate
16	Ozlu, et al. (2021) [25]	Randomized controlled study	67 COVID-19 patients	PMR exercises twice a day for 5 days	Statistically significant reductions in anxiety and improvements in sleep quality (*p* < 0.05)	PMR can effectively reduce anxiety and improve sleep quality in COVID-19 patients	6/10	Moderate
17	Hajibashi, et al. (2023) [26]	Randomized controlled trial	Discharged COVID-19 patients (*n* = 52)	Pulmonary telerehabilitation (PTR) + PMR vs. PTR alone for 6 weeks	Significant improvements in sleep quality, fatigue, and anxiety in the PMR + PTR group (*p* < 0.05)	PMR enhances pulmonary rehabilitation outcomes in post-COVID recovery	9/10	Low
18	Saleh, et al. (2024) [26]	Randomized controlled trial	146 COVID-19 survivors in Jordan	PMR for 30 min/day for 5 days; assessed anxiety (STAI) and sleep quality (SRSS)	Significant improvement in sleep quality (*p* < 0.05); reduced anxiety (*p* < 0.05)	PMR is effective in improving sleep and reducing anxiety in COVID-19 survivors	6/10	Moderate
19	Maritescu, et al. (2024) [4]	Randomized controlled trial	61 post-COVID-19 patients	Pulmonary Rehabilitation (PR) with PMR for 21 days vs. PR alone	PMR + PR group had greater improvements in anxiety (GAD-7), sleep quality (PSQI), and mental health (GHQ-12) (*p* < 0.0001)	Adding PMR to PR enhances mental health outcomes in post-COVID-19 patients	7/10	Moderate
20	Herdiman H., et al. (2022) [27]	Quasi-experimental study	COVID-19 patients in Bandung	PMR for 20–30 min twice daily for 5 days	Significant reductions in anxiety and improved sleep quality in the intervention group (*p* < 0.05)	PMR is effective in reducing anxiety in hospitalized COVID-19 patients	7/8	Moderate
21	Seid A.A., et al. (2023) [28]	Systematic review and meta-analysis	COVID-19 patients	Analysis of PMR effects on anxiety, depression, sleep quality, and quality of life	PMR interventions significantly reduced anxiety and improved sleep quality, though long-term effects remain uncertain (*p* < 0.05)	PMR can be a useful short-term intervention for COVID-19 patients	10/10	Low
22	Xiao C.X., et al. (2020) [29]	Observational study	COVID-19 patients	PMR training vs. routine care	Significant improvements in anxiety and sleep quality in the intervention group (*p* < 0.05)	PMR should be clinically promoted for anxiety and sleep issues in COVID-19 patients	7/8	Moderate
23	Liu K., et al. (2020) [30]	Randomized controlled trial	COVID-19 patients	PMR for 30 min/day for 5 days	Significant reductions in anxiety (*p* < 0.001) and improved sleep quality	PMR is a feasible and effective auxiliary method for improving mental health in COVID-19 patients	6/10	Moderate
24	Ganjeali S., et al. (2022) [31]	Randomized clinical trial	Nurses caring for COVID-19 patients	Demonstration-based PMR training	Significant reductions in stress and anxiety in the intervention group (*p* < 0.001)	PMR should be included in nursing training to improve mental well-being	9/10	Low
25	Maritescu, et al. (2025) [32]	Randomized controlled trial	22 adult patients with cystic fibrosis (mean age ≈24 years)	PMR 3×/week for 4 weeks added to standard pulmonary rehabilitation (PR); evaluated with HADS, PSQI, CFQ-R, 6MWT, FEV_1_	Significant reduction in anxiety (*p* = 0.05) and depression (*p* = 0.02); sleep quality improved (*p* < 0.01); no significant changes in FEV_1_ or 6MWT	PMR added to pulmonary rehabilitation improves mental health and sleep quality in adults with cystic fibrosis and may complement standard care	7/10	Moderate
26	Enita Dewi, et al. (2022) [33]	Pre-experimental	26 adult asthma patients	10-min PMR post-admission; anxiety measured (BAI)	Severe anxiety dropped to 0%, mild anxiety rose to 57.69% (*p* = 0.001)	PMR significantly reduces anxiety in acute asthma; applicable in ER	9/10	Moderate
27	Georgia Georga, et al. (2018) [34]	Randomized controlled trial	42 asthma patients	8-week SM program (PMR + biofeedback-assisted breathing)	Significant improvements in stress, QOL, and asthma control (*p* < 0.0001)	PMR + breathing training improves asthma-related outcomes	7/10	Moderate
28	Syafa’atun Mirzanah, et al. (2020) [35]	Review article	Patients with chronic diseases	Review of 6 studies on PMR for sleep quality	All showed significant improvement in sleep quality; Sleep improved from 10.5 ± 2.4 to 5.8 ± 1.7; *p* < 0.05	PMR is effective in enhancing sleep quality in chronic disease	10/10	Low
29	Yunping Li, et al. (2015) [36]	Randomized controlled trial	130 PAH patients	12 weeks of group + home PMR vs. stretching	Significant improvement in anxiety, depression, QOL (*p* < 0.05)	PMR enhances mental health in PAH patients	8/10	Moderate
30	Ratna Sari Dinaryanti, et al. (2019) [37]	Quasi-experimental	19 lung cancer patients with dyspnea	PLB + PMR twice daily for 6 days	Improved oxygen saturation and relaxation (*p* < 0.05)	Breathing and PMR exercises are beneficial for lung cancer dyspnea	5/6	Moderate
31	Aksu, et al. (2017) [38]	Randomized controlled trial	26 post-pulmonary resection patients	PMR twice daily for 7 days	Improved sleep, QOL; reduced pain vs. control (*p* < 0.05)	PMR supports recovery after pulmonary resection	7/10	Moderate
32	Lin, et al. (2023) [39]	Randomized controlled trial	140 thoracic surgery patients	PMR tailored to respiratory muscle assessment vs. routine care	Better pulmonary function, sputum excretion, QOL (*p* < 0.05)	PMR enhances post-op recovery in thoracic surgery	5/10	Moderate

PMR—Progressive Muscle Relaxation; PLB—Pursed-Lip Breathing; PSQI—Pittsburgh Sleep Quality Index; FACIT-T—Functional Assessment of Chronic Illness Therapy—Fatigue Scale; BDI II—Beck Depression Inventory II; HADS—Hospital Anxiety and Depression Scale; GHQ-12—General Health Questionnaire; CFQ-R—Cystic Fibrosis Questionnaire—Revised; 6MWT—6-Minute Walk Test; FEV1—Forced Expiratory Volume in 1 Second; STAI—State-Trait Anxiety Inventory; SRSS—Sleep Rating Scale System; QOL—Quality of Life; BAI—Beck Anxiety Inventory; SM program—Self-management Program; DVD—Digital Versatile Disc; PAH—Pulmonary Arterial Hypertension; RCT—Randomized Controlled Trial.

## Data Availability

The supporting data for the findings of this study can be obtained by contacting the corresponding author upon request. However, the data cannot be publicly accessed due to privacy and ethical considerations.

## Abbreviations

The following abbreviations are used in this manuscript:

PMR	Progressive Muscle Relaxation
COPD	Chronic Obstructive Pulmonary Disease
CF	Cystic Fibrosis
COVID-19	Coronavirus Disease 2019
PAH	Pulmonary Arterial Hypertension
FEV_1_	Forced Expiratory Volume in 1 s
FVC	Forced Vital Capacity
HRV	Heart Rate Variability
EEG	Electroencephalogram
HPA	Hypothalamic–Pituitary–Adrenal axis
SpO_2_	Peripheral Oxygen Saturation
HADS	Hospital Anxiety and Depression Scale
GHQ-12	General Health Questionnaire-12
PSQI	Pittsburgh Sleep Quality Index
SRSS	Sleep State Self-Rating Scale
STAI	State-Trait Anxiety Inventory
GAD-7	Generalized Anxiety Disorder-7
PHQ-9	Patient Health Questionnaire-9
6MWT	6-Minute Walk Test
CASIS	COPD and Asthma Sleep Impact Scale
CAFS	COPD and Asthma Fatigue Scale
BAI	Beck Anxiety Inventory
ACQ	Asthma Control Questionnaire
AQLQ	Asthma Quality of Life Questionnaire
SGRQ	St. George’s Respiratory Questionnaire
SAS	Self-Rating Anxiety Scale
SDS	Self-Rating Depression Scale
SF-36	Short Form Health Survey
ARAS	Ascending Reticular Activating System
RCT	Randomized Controlled Trial
JBI	Joanna Briggs Institute
PTR	Pulmonary Telerehabilitation
LMRT	Laura Mitchell’s Relaxation Technique
JPRT	Jacobson’s Progressive Relaxation Technique
GDB	Guided Deep Breathing
MBS	Modified Borg Scale
mMRC	Modified Medical Research Council
CI	Confidence Interval
SMD	Standardized Mean Difference
